# Welfare implications on management strategies for rearing dairy calves: A systematic review. Part 2 – Social management

**DOI:** 10.3389/fvets.2023.1154555

**Published:** 2023-04-17

**Authors:** Patricia Carulla, Arantxa Villagrá, Fernando Estellés, Isabel Blanco-Penedo

**Affiliations:** ^1^Instituto de Ciencia y Tecnología Animal, Universitat Politècnica de València, Valencia, Spain; ^2^Centro de Tecnología Animal, Instituto Valenciano de Investigaciones Agrarias, CITA-IVIA, Segorbe, Castellón, Spain; ^3^Departamento de Ciencia Animal, Universidad de Lleida, Lleida, Spain; ^4^Department of Clinical Sciences, Swedish University of Agricultural Sciences, Uppsala, Sweden

**Keywords:** rearing calves, Holstein calves, welfare, social management, animal production, dairy sector

## Abstract

**Introduction:**

Raising a healthy calf up to puberty is essential for optimal farm performance. It is therefore, it is necessary to promote animal welfare from the three spheres during this short period. Social management has been postulated as essential in lowering stress and consequently improving calf welfare during this period. Only the health sphere has been studied for a long time, but more recent studies have recently promoted positive experiences and emotional states from affective states or cognitive judgment and natural living spheres. A systematic review of different management strategies in rearing dairy calves according to the three spheres of animal welfare has been conducted using an electronic search strategy.

**Methods:**

The analysis and extraction of information from the studies were performed according to a protocol. From 1,783 publications screened, only 351 met the inclusion criteria.

**Results:**

The publications identified in the search can be divided into two main groups, feeding and social management, based on the main topic of the publication. This review provides an overview of social management, understood as the calf’s interaction with others around it.

**Discussion:**

Primary social management issues that emerged were social housing with congeners, separation from the mother and human-animal interaction, distributed in the three broad spheres of animal welfare. The review highlights unresolved questions about how social management practices affect the three spheres of animal welfare at this life stage and the need to standardize good socialization practices for this stage. In conclusion, all the information shows that social housing has improved animal welfare from affective states, cognitive judgment, and natural living spheres. However, gaps in research were identified in relation to the optimal time to separate the calf from the mother, the optimal time to group with conspecifics after birth and group size. Further research on positive welfare through socialization are needed.

## 1. Introduction

Infancy is one of the most important periods of development for mammals, with the environment playing a crucial role (1). In the case of calves, welfare in the early stages of life is one of the most challenging tasks on a dairy farm. Ensuring the best welfare from all three spheres (biological functioning and animal health, affective states or cognitive judgement, and natural living) during the rearing period has a direct influence on calf development and maybe a possible preventive solution to future problems (2). Furthermore, there are also regulations that set minimum standards for the protection of calves, which can be used as a guideline for rearing animals (3).

The recent development of not only avoiding negative experiences but also seeking positive ones (4–6), coupled with consumer demand, has led to increased socialization studies (7). Social interactions have been studied, from maternal bonding to interaction with humans and conspecifics.

Bonding with the mother and the best time for separation still need further study (8). Traditionally, dairy calves are separated from their dam within hours after birth and reared artificially, but in recent years cow-calf contact rearing has received more attention as a more natural system (9, 10).

Furthermore, a good relationship between cattle and humans is important as it allows for reduced stress responses to routine management practices, thus improving welfare (11). The quality of the human-animal relationship plays an essential role in defining the welfare of the animals (9) and, unquestionably, in dairy animals the human-animal interactions are more frequent and more intensive than in the other farm. From birth to adulthood calf are in contact with humans as some procedures are performed daily such as milking (11).

Finally, social interaction between calves is very important, as cattle are a predatory and gregarious species and being together is an important safety factor for them (12). Current research shows the benefits of rearing calves in pairs or small groups. Housing calves with at least one other calf can improve consumer perception, animal welfare, calf growth (7) and cognitive development (1). Although many welfare improvements have been seen, the effect on health is less clear (7), but the future trend is towards social housing.

Therefore, the second part of this systematic review (2) aims to provide a detailed overview of the different social management practices and their impact on the welfare of preweaned calves. In addition, this review aimed to identify gaps in knowledge for further research.

## 2. Materials and methods

The systematic review protocol is described in detail in the first part of this systematic review (2).

### 2.1. Search terms and search strategies

The aim of this search was to identify social management strategies and analyze their effect on the welfare of preweaned calves. The search terms and strategy are available elsewhere (2). In addition, relevant references found during the update and review process have been included in the manuscript.

### 2.2. Data extraction

A data extraction form and screening process was developed for this systematic review, which is available elsewhere (2). As mentioned in the first part, the publications were clustered according to: colostrum, milk replacer, started feed, weaning, separation from mother, animal-human interaction and interaction with congeners. The last three groups will be developed in this review.

## 3. Results

### 3.1. Synthesis of results of social management

Several studies have analyzed the effects of social management techniques on early calf development, particularly in preweaned calves. All the different social strategies are examined in a disaggregated way compared to the framework of the three welfare spheres.

**Figure 1:**
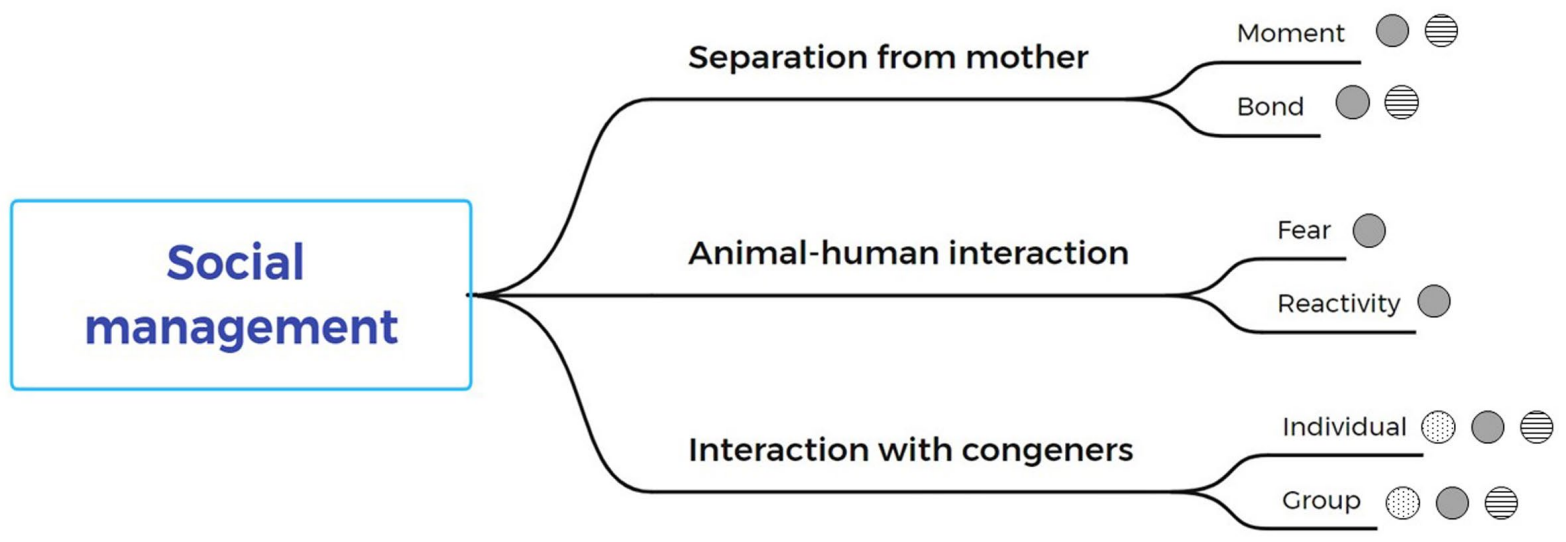
Critical points of each phase of social management and their relationship to animal welfare spheres. Dotted spheres represent biological functioning and health, spheres with horizontal lines relate to natural living, and gray spheres relate to affective states or cognitive judgments.

Social management practices were broadly described in the search as practices that affect animal welfare. Calves are gregarious animals, so social management greatly impacts their welfare. Under the umbrella of social management, shown in Figure 1, separation from mother, human-animal interaction and conspecific interaction are assessed and analyzed.

## 4. Discussion

Although the neonatal and infant periods are important for adequate physical, behavioural, and cognitive development into adulthood, literature reviews over the past two decades have resulted in many publications on different management strategies, but few studies addressing the three spheres of animal welfare. However, there has been a shift in approaches to animal welfare assessment to include animal-based indicators related to emotional state and natural life. The application of this new welfare knowledge will improve the daily lives of animals.

Management has been shown to significantly influence the welfare of preweaned calves. The first part, looked at feeding management (2), so in this part, we will look at social management.

### 4.1. Social management for welfare

Social management was found to be critical in the early stages of calf development. As shown above, in Figure 1, social management practices such as (a) separation from the mother (13), (b) human-animal interaction (14), and (c) conspecific interaction with their congeners (15) have been widely described in the reviewed literature as practices that affect animal welfare. In the case of conspecific interaction, individual and group housing of different sizes have been studied. The level of socialization affects the sphere of affective states or cognitive judgement and natural life and, to a lesser extent, the sphere of biological functioning and health.

#### 4.1.1. Separation from the mother

One of the most common practices in dairy farming is to separate calves from their mothers immediately after calving (16). However, consumers question the ethics of this practice (17), and calf rearing with cow contact has increased in recent years (8).

Leaving calves with their mothers can have health and psychological benefits. Calves gain more weight and have fewer diseases (13), have more favorable emotional states (17), and show more playful behaviour (18). However, the longer the calf separated from its mother, the stronger the bond is formed and therefore the more negative the behavioural response after separation (13, 19).

In addition to separation time, other separation strategies have also been investigated (8, 20, 21). There are four main rearing systems described in the literature. Firstly, full contact systems where cow and calf have unrestricted access to each other. Second, partial contact systems which can be implemented in a variety of ways: with restricted suckling systems, where there is brief contact only for suckling; with half day contact systems, where the cow and calf are housed together during the day or night, and finally with cow systems, where a cow suckles 2 to 4 calves, usually without milking (10).

With regard to full contact, some “anti-suckling devices” have been used; such as nose rings or nose flaps, although this device allows the calf to be with the mother, it seems to cause frustration. Wenker et al. (21) reported that animals with full contact but nose-flap were more stressed than those with partial contact. In addition, the combination between full contact and abrupt weaning stresses cows and calves (20). Partial contact, on the other hand, has been shown to reduce abnormal behaviour such as cross-sucking and no differences in health have been found (8). Half-day contact seems particularly promising as animals become accustomed to separation, experience positive humane treatment and calves can learn to use a milk feeder to prevent stunting after weaning (10). All of these strategies have a direct effect on affective states or cognitive judgment (17).

Although Nicolao et al. (20) have shown that the best compromise between cow milk yield and calf welfare is a long period of cow-calf contact between the morning and evening milking, more research is needed to investigate strategies to improve the process of debonding and weaning.

#### 4.1.2. Animal-human interaction

Establishing good human interaction improves welfare (9) and reduces animal fear and distress from affective states or cognitive judgement. Workers without training or low job satisfaction elicit higher responses in the avoidance test, which is why calves are more fearful of them (14). Poor management affects the approach distance of calves to humans as the reactivity is due to the constant stress of poor management. The animal has also been shown to maintain this response. Human contact on farms is a very important factor to be consider and has been little studied, probably because of the complexity of the assessment (22). More research is needed in this area.

#### 4.1.3. Interaction with congeners

Finally, housing and social interaction with other calves significantly impact animal welfare in terms of social management. Animals can be housed individually, in pairs, or groups, although individual housing is the most common practice. It has been shown that calves housed individually have consistent behavioural and developmental deficits. In contrast, social housing, whether in pair-housed or group-housed, has been shown to improve production rates through grain consumption and grow as well as or better than individual housing (7), in addition, to improving cognitive, and behavioural parameters related to affective states or cognitive judgement and natural living (1).

Although individually housed calves have complete control over their health and food intake, the literature shows that they have deficits at the level of affective states or cognitive judgement and natural living. Calves deprived of contact with other animals show greater anxiety responses to the environmental novelty test (15, 23, 24), greater anxiety when encountering other calves (25), cognitive and learning deficits (26, 27), and play deprivation (28).

In the case of pair-housing, the optimal time to put the animals together after birth is still under investigation. However, in the available studies, have found no difference between pair-housing immediately after birth or at 3 weeks of age (29, 30). Animals have better productive parameters than when housed individually: they consume more solid feed (24, 31, 32), they have a higher average daily gain both before (1, 29, 33) and after weaning (34) and they achieve a higher weight at weaning (35). When calves are housed in pairs, their welfare is improved in terms of biological functioning and health.

Studies on the effects of social housing on health, studies are controversial. On the one hand, some studies show an increased risk of disease (36, 37), while other studies have shown no risk to the health of socialized calves (25, 38, 39).

The most significant difference in welfare between calves’ pair-housed and individual housed calves in behavioural responses, affecting spheres of affective states or cognitive judgement and natural life. In the studies reviewed, it is clear that social housing provides a greater opportunity for natural social behaviours, and animals are more exercised (33). Calves spend more time resting with their partner (35, 40), improve their affective appearance (41), show less stress, and increase their motivation to play (42). It is well known that when the animals are healthy, they are more motivated to play (43) whereas when they are sick they spend more time lying down and eat less (44). These behaviours may therefore be useful in detecting health problems. In terms of responses to tests of novel social and environmental situations, paired calves have been shown to be less reactive and more curious (23, 25). In addition, this type of housing may alleviate some of the negative aspects of weaning, as this stress is cushioned by social support (38, 45, 46), and less non-nutritive oral behaviours are observed (34). Finally, calves housed in pairs are better prepared to live in groups after weaning (47).

However, studies have shown that pair-housing also has its limitations. In social housing, competition for feed and cross-sucking problems have been reported (48). Cross-sucking is a welfare problem defined as sucking on any part of the body of the calves in the same pen and it can lead to abscesses in the ears and belly button (33, 49, 50). An excellent way to reduce this behaviour is to offer the milk with slow-flow nipples (51) or with anti-sucking devices (52), while competition can be avoided by using long barriers that occupy the front half of the calf during feeding (48).

Calves have been shown to change their behaviour to accommodate mates (40) and to display more natural behaviour (53, 54) when they are with their peers. There is currently, a lot of interest in taking social housing for calves a step further by housing them in group. In this case, there must be an equal or greater number of teats than animals in the group because otherwise, competitiveness increases and feeding time decreases (55, 56). Introducing new precision livestock farming technologies can facilitate this type of housing, as automatic feeders or remote monitoring systems improve individual attention and save labor, even for grouped calves (15, 57).

## 5. Conclusion

There is currently no clear agreement on all issues relating to calf social management strategies and their impact on welfare. An understanding of welfare issues by management can help prevent future problems. From all the information reviewed, the most important gaps in knowledge are the optimal time to separate the calf from its mother, and further research into the positive welfare benefits of socialization with humans and congeners. Collaboration between scientific research and the dairy sector is essential to establish management standards that support proper growth, ensure health and welfare, and facilitate weaning.

## 6. Implications

This paper provides an overview of the social management strategies used in the rearing of Holstein calves and how this management affects the three spheres of animal welfare. Understanding the influence of management on welfare helps to prevent future problems.

Based on the information reviewed, some recommendations can be summarized to optimize the social management of calves. Separation from the dam should occur immediately after birth. In addition, good human-animal interaction is essential to implement. In terms of socialization with conspecifics, housing in pairs or groups immediately after birth improves animal welfare.

In addition, the authors have produced a table (Supplementary Table S1), suggesting different management practices and their impact on each of the spheres of animal welfare and the missing gaps that need to be investigated in the future.

## Data availability statement

The raw data supporting the conclusions of this article will be made available by the authors, without undue reservation.

## Author contributions

PC, AV, FE, and IB-P contributed to the conception and design of the study. PC and IB-P organized the database. PC wrote the first draft of the manuscript. All authors contributed to the article and approved the submitted version.

## Funding

This research was supported by the Centro para el Desarrollo Tecnológico Industrial and Cowvet S.L through project CDTI-IDI-20200936. In addition, PC held a grant from the Ph.D. student’s research program of the Universitat Politècnica de València (PAID-01-20).

## Conflict of interest

The authors declare that the research was conducted in the absence of any commercial or financial relationships that could be construed as a potential conflict of interest.

## Publisher’s note

All claims expressed in this article are solely those of the authors and do not necessarily represent those of their affiliated organizations, or those of the publisher, the editors and the reviewers. Any product that may be evaluated in this article, or claim that may be made by its manufacturer, is not guaranteed or endorsed by the publisher.
