# Supplementary material for: Welfare implications on management strategies for rearing dairy calves: A systematic review. Part 2 – Social management
Source: Front Vet Sci. 2023 Apr 17;10:1154555. doi: 10.3389/fvets.2023.1154555 (PMC10150551; doi:10.3389/fvets.2023.1154555)
Supplement: Supplementary file 1 [file Table_1.docx]

Table S.1. Relationship between the different management practices and their impact on each animal welfare sphere. The symbol “-“ refers to a lack of information on how the management practice affects welfare. The symbol “!” indicates to missing gaps that need to be investigated in the future

|  |  | Three spheres of animal welfare | | |
| --- | --- | --- | --- | --- |
|  | Practice management | Biological functioning and health | Affective states or cognitive judgment | Natural living |
| SEPARATION MOTHER | Separation from mother immediately | Gain less weight | There is no yet affective relationship | No differences in behaviour |
|  | Separation from mother longer | Gain more weight and have less disease | Before better mood | After negative behavioural |
| HUMAN | Good human-animal interaction | ! | Reduce fear and anguish | ! |
|  | Bad human-animal interaction | ! | Increase fear-anguish | ! |
| CONGENERS | Individual housing | Control of their health | Cognitive deficits and more fear | More non-nutritive oral behaviour |
|  | Social housing | Better productive parameters  ! | Less reactive to novelty  More curious | More natural behaviour, exercise, play |
